# Avoidable Benign Kidney Tumor Resections—Data from a Tertiary Care Cancer Institute

**DOI:** 10.15586/jkcvhl.v11i4.286

**Published:** 2024-10-01

**Authors:** Arun Ramdas Menon, Vivek Patel, Nivedita Suresh, Anand Raja

**Affiliations:** 1 Department of Surgical Oncology, Cancer Institute (WIA), Chennai, India;; 2 Department of Onco-Pathology, Cancer Institute (WIA), Chennai, India

**Keywords:** benign kidney tumor, kidney cancer, nephrectomy, overtreatment, renal tumor

## Abstract

Enhancing renal masses are conventionally treated as malignant unless proven otherwise due to the difficulty distinguishing between malignant and benign tumors based on imaging. Data from the Western registries suggests overtreatment of renal tumors with a Benign Kidney Tumor Resection Rate (BKTRR) ranging from 10 to 33%, with an increasing trend. Since robust, population-based data from India was unavailable, we sought to determine BKTRR in an apex cancer institute, which would provide insight into the rates in the community. The institutional kidney tumor database was queried for all patients aged ≥18 years with renal neoplasms between January 2000 and December 2022. Patients who underwent surgery, either radical or partial nephrectomy, with intent to cure were analyzed and the BKTRR during the study period was evaluated. A total of 330 patients underwent surgery for renal tumors presumed to be malignant. A final pathologic diagnosis of the benign tumor was made in 16 (4.8%) patients, comprising 7.2, 7.2, and 3.7% of resections with LTD ≤4, 4–7, and >7 cm, respectively. Asymptomatic benign tumors ≤7 cm comprised 3.0% of all resections, and these were potentially unnecessary surgeries. A multivariable analysis suggested that no patient or imaging characteristic could predict a final benign extirpative pathology. Our study suggests a lower rate of BKTRR compared to the published international literature but is likely to be the lower limit of that in the community. Population-based studies are required to determine the true BKTRR and the quantum of potentially unnecessary surgeries for benign kidney tumors.

## Introduction

All enhancing renal masses are conventionally considered malignant unless proven otherwise and are treated as such, usually with extirpative surgery or ablation. This stems from the inherent difficulty in distinguishing malignant versus benign tumors based on imaging (1).

Consequent to this approach, data from the Western surgical series suggests an overtreatment of renal tumors with a Benign Kidney Tumor Resection Rate (BKTRR) ranging from 10 to 33% (2–5). An increasing trend of BKTRR has also been reported, which has been attributed to increased detection of renal masses due to the widespread use of cross-sectional imaging (3, 4). The surge in adoption of robotic surgery has also fueled more aggressive treatment of small renal masses (SRMs), 20–30% of which are benign (6).

Surgery for an asymptomatic benign renal tumor constitutes an unnecessary surgical procedure that has negative impacts on both the patient as well as the healthcare system (7, 8). However, there is a lack of population database studies on BKTRR in the Indian healthcare scenario, and extrapolating Western data may be inaccurate due to intrinsic epidemiological differences in renal cell carcinoma (RCC) in India compared to the West (9).

Our institution is an apex cancer center in South India, with a patient base comprising referrals from other hospitals due to tumor complexity and anticipated difficult surgery. We hypothesized that due to the aforementioned significant referral bias, our benign kidney tumor resection rate must be low.

## Methods

This work was conducted at the Cancer Institute (WIA), Chennai, India.

After obtaining approval from the Institutional Review Board, the prospectively maintained institutional kidney tumor database was retrospectively reviewed for all patients aged ≥18 years, who presented with renal neoplasms between January 2000 and December 2022. Pertinent clinicopathologic data was abstracted, including stage at presentation, management elected, and pathologic outcomes. Patients who underwent surgery, either radical or partial nephrectomy, with the intent to cure were analyzed and the BKTRR during the study period was evaluated.

Statistical analyses were conducted in SAS v9.4 (Cary, NC) at a significance level of 0.05. Patient and tumor characteristics were described using mean, median, and interquartile ranges (IQR) for continuous variables, and frequencies for categorical variables. Pathologic classification and staging were based on the World Health Organization Classification of Renal Tumors (10) and the American Joint Committee on Cancer Staging System (11), respectively. Comparisons between benign and malignant extirpative pathology were made using the Mann–Whitney U test for continuous variables and Pearson’s chi-square test for categorical variables. Multivariable Cox regression models were generated to evaluate the association between benign pathology, patient, and tumor characteristics.

Overall, the study aims to provide insight into the BKTRR in the Indian healthcare scenario and the potential impact of referral bias on the data.

## Results

Of the 464 renal tumors treated at our institute over the study period, 117 (25.2%) presented with histologically proven metastatic disease (20.5%) or with inoperable disease (4.7%) and were excluded from the analysis. Furthermore, 17 (3.7%) patients did not undergo surgery due to poor performance status or patient refusal. No patient in our registry underwent active surveillance or ablation.

A total of 330 patients underwent surgery for renal tumors, presumed to be malignant, with intent to cure; and 282 of these were radical nephrectomies and 48 were partial nephrectomies (Table 1).

**Table 1:** Patient and tumor characteristics.

	Total (N = 330)	Benign (N = 16)	Malignant (N = 314)	P
**Age**, years; median, mean (IQR)	54.0 (52.8, 45.0–61.0)	53.0 (51.2, 45.5–55.0)	54.5 (52.9, 18.0–84.0)	0.588
**Gender**, n (%)				
Male	186 (56.4)	6 (37.5)	180 (57.3)	0.119
Female	144 (43.6)	10 (62.5)	134 (42.7)	
**Laterality**, n (%)				
Right	168 (50.9)	6 (37.5)	162 (51.6)	0.502
Left	160 (48.5)	10 (62.5)	150 (47.8)	
Bilateral	2 (0.6)	0 (0.0)	2 (0.6)	
**Presentation**, n (%)				
Asymptomatic	91 (27.6)	7 (43.8)	84 (26.8)	0.137
Symptomatic	239 (72.4)	9 (56.3)	230 (73.2)	
Abdominal pain	133 (40.3)	6 (37.5)	127 (40.4)	0.253
Hematuria	105 (31.8)	1 (6.3)	104 (33.1)	
Mass abdomen	14 (4.2)	1 (6.3)	13 (4.1)	
Weight loss/fatigue	20 (6.1)	0 (0.0)	20 (6.4)	
**LTD**, cm; median, mean (IQR)	7.0 (8.1, 4.5–10.0)	5.3 (8.2, 3.9–11.1)	7.0 (8.1, 4.5–10.0)	0.608
**Procedure**, n (%)				
Radical nephrectomy	282 (85.5)	13 (81.3)	269 (86.7)	0.624
Partial nephrectomy	48 (14.5)	3 (18.8)	45 (14.3)	

IQR, interquartile range; LTD, longest tumor diameter.

The median (mean, IQR) patient age was 54.0 years (52.8, 45.0–61.0), and 43.6% were females. 27.6% of patients had incidentally diagnosed tumors. The median (mean, IQR) longest tumor diameter (LTD) was 7.0 cm (8.1, 4.5–10.0).

A final pathologic diagnosis of benign tumor was made in 16 (4.8%) patients. Benign tumors comprised 7.2, 7.2, and 3.7% of resections with LTD ≤4, 4–7, and >7 cm (Table 2). Asymptomatic benign tumors ≤7 cm comprised 3.0% of all resections. Five (31.3%) benign tumors had LTD >10 cm.

**Table 2:** Distribution of tumors based on size and pathologic stage.

LTD (cm); n (%)	Total (n = 330)	Benign (n = 16)	Malignant (n = 314)
**<2.0**	**9 (2.7)**	**0 (0.0)**	**9 (2.9)**
2.1–3.0	21 (6.4)	2 (12.5)	19 (6.1)
3.1–4.0	39 (11.8)	3 (18.8)	36 (11.5)
**≤ 4.0**	**69 (20.9)**	**5 (31.3)**	**64 (20.4)**
4.1–5.0	39 (11.8)	3 (18.8)	36 (11.5)
5.1–6.0	29 (8.8)	1 (6.3)	28 (8.9)
6.1–7.0	30 (9.1)	1 (6.3)	0 (0.0)
**4.1 to ≤7**	**98 (29.7)**	**5 (31.3)**	**93 (29.6)**
7.1–8.0	32 (9.7)	0 (0.0)	32 (10.2)
8.1–9.0	26 (7.9)	0 (0.0)	26 (8.3)
9.1–10.0	23 (7.0)	1 (6.3)	22 (7.0)
**7.1**–**10**	**81 (24.5)**	**1 (6.3)**	**80 (25.5)**
**>10.0**	**80 (24.2)**	**5 (31.3)**	**75 (23.9)**
NA	2 (0.6)	–	2 (0.6)
**Pathologic T Stage; n (%)**			
**pT1a**	59 (17.9)	–	59 (18.8)
**pT1b**	86 (26.1)	–	86 (27.4)
**pT2a**	55 (16.7)	–	55 (17.5)
**pT2b**	27 (8.2)	–	27 (8.6)
**pT3a**	50 (15.2)	–	50 (15.9)
**pT3b**	18 (5.5)	–	18 (5.7)
**pT3c**	5 (1.5)	–	5 (1.6)
**pT4**	11 (3.3)	–	11 (3.5)
**NA**	3 (0.9)	–	3 (1.0)

LTD, longest tumor diameter; NA, not available.

Angiomyolipomas (AMLs) and renal oncocytomas (ROs) were the most common, accounting for 37.5 and 31.2% of all resected benign tumors. Other benign lesions accounted for 31.2% (Table 3).

**Table 3:** Clinicopathologic features of all resected benign tumors.

No	Year of Surgery	Age (years)	Sex	Presentation	LTD (cm)	Laterality	Pathology	RN/PN
1	2003	65	Male	Incidental	5.5	Right	Oncocytoma	RN
2	2003	38	Female	Abdominal pain	9.5	Left	Angiomyolipoma	RN
3	2004	55	Female	Abdominal pain	5.0	Right	Adrenocortical hyperplasia with adrenal hemangioma	RN
4	2005	55	Female	Incidental	3.5	Left	Oncocytoma	RN
5	2005	74	Female	Abdominal pain	13.0	Left	Angiomyolipoma	RN
6	2005	49	Female	Abdominal pain	5.0	Right	Oncocytoma	RN
7	2007	37	Female	Abdominal pain	15.0	Left	Leiomyoma	RN
8	2007	54	Male	Incidental	3.0	Left	Lipoma	PN
9	2011	35	Male	Abdominal pain	4.0	Right	Angiomyolipoma	PN
10	2011	49	Male	Incidental	5.0	Left	Angiomyolipoma	RN
11	2011	52	Female	Incidental	20.0	Left	Oncocytoma	RN
12	2015	55	Female	Incidental	3.4	Right	Pyelonephritis	RN
13	2019	57	Female	Incidental	7.0	Right	Oncocytoma	RN
14	2022	54	Male	Hematuria	10.5	Left	Angiomyolipoma	RN
15	2022	46	Male	Mass abdomen	20.0	Left	Cystic nephroma	RN
16	2022	44	Female	Incidental	2.2	Left	Angiomyolipoma	PN

LTD, longest tumor diameter; PN, partial nephrectomy; RN, radical nephrectomy.

Of the 314 patients with malignant renal tumors, the median (mean, IQR) LTD was 7.0 (8.1, 4.5–10.0), and 113 (35.3%) of them had stage ≥ pT2b.

**Figure 1:**
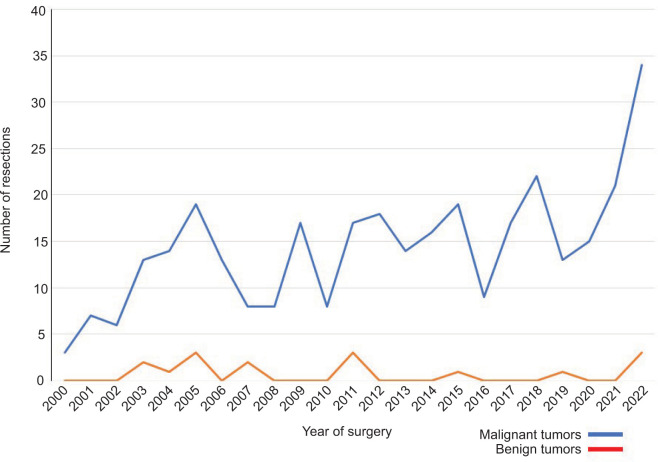
Trend in benign resections over the 23-year study period. Graph depiction of the number of benign (red) and malignant (blue) resected kidney tumors per year during the 23-year study period.

No significant trend was noted in the rate of benign resections per year (mean/range [%] = 3.3/0–15.8; Figure 1).

On multivariable analysis, we did not identify any significant patient or imaging characteristic that could predict a final extirpative pathology of a benign kidney tumor (Table S1).

## Discussion

In the absence of robust population-based data from India, our study evaluated the BKTRR in an apex cancer institute and attempted to gauge the quantum of potentially avoidable benign kidney tumor resections. Our BKTRR of 4.8% is lower than that reported in the Western series, where benign resections account for 15–30% of all resected tumors (2–4). Rates of 4.4–11% have been reported from other Asian countries (12–14). In our series, asymptomatic benign tumors ≤7 cm accounted for 3% of the overall resections, and these may potentially have been unnecessary surgeries. A significant proportion of patients presenting with symptoms, rather than an incidental diagnosis, larger median tumor size of resected tumors, with over a third of patients presenting with ≥ pT2b disease, and considerable patients presenting with inoperable or metastatic disease reflect our significant referral bias, and attribute to the lower BKTRR.

Surgery for an asymptomatic, benign renal tumor is of concern due to its adverse consequences on both the patient and the healthcare system (7). The surgical morbidity of a radical or partial nephrectomy is not trivial, with reported complication rates of 14 and 20%, respectively (15). Clavien Dindo Grade 3–4 complications in 2.5–6% (15, 16) and perioperative mortality rates of 0.5–1% in 200 patients have been reported (15, 17), with one study indicating a greater risk with benign tumors (8). The consequences of nephrectomy on decreasing overall renal function, with long-term adverse effects on cardiovascular health and longevity, have been well reported (18).

Johnson et al. reported an 82% increase in nephrectomies for benign lesions between 2000 and 2009, with an estimated annual 6000 unnecessary nephrectomies for benign tumors (2). The annual cost of managing benign tumors in the United States has been estimated to be in the region of 153 million dollars (8). Data from India is sparse and limited to small single institutional series. Additionally, costs related to surgery are difficult to estimate due to heterogenicity in healthcare systems; however, the public health magnitude of this intuitively seems high. Avoiding unnecessary surgery can alleviate overburdened healthcare systems, and spare patients the associated financial toxicity.

Benign resection rates from Japan, South Korea, and China have been reported to be between 4.4 and 11% (12–14). The lower rates have been attributed to lower incidence of oncocytomas, variations in extirpative pathology evaluation and reporting, particularly of oncocytic tumors, less intense use of cross-sectional imaging, and more liberal use of repeat or advanced imaging in characterizing indeterminate lesions (1, 2, 13).

The association of size and likelihood of benign pathology has been a matter of controversy with conflicting evidence in the literature (3, 4). Although our rates of benign pathology decreased with increasing tumor size, this was not statistically significant. Interestingly in our series, no tumor <2 cm was benign. Additionally, 3.7% of tumors >7 cm were benign, and five benign tumors were >10 cm. This is likely to be a result of our referral bias as more complex, and larger tumors are likely to have been referred to our institution due to anticipated surgical difficulties.

We did not find any statistically significant difference in patients or tumor imaging characteristics among benign and malignant renal tumors, likely due to our relatively small cohort, and skewed referral patterns. The multivariable analysis did not identify any analyzed patient or tumor characteristic that could predict benign histology. Other authors have variably reported female gender, asymptomatic presentation, smaller size, low tumor complexity or nephrometry score, lower body mass index (BMI), and lower serum creatinine to be associated with benign pathology (5, 13, 14, 19–23). These parameters are likely predictive for benign tumors only in their respective study cohorts, and may not be generalizable. In addition, although they may help in counseling a patient regarding the likelihood of benign resection, they are not useful to conclusively establish a benign diagnosis preoperatively. Lane et al. developed a nomogram, using patient age, CT size, local symptoms at diagnosis, and history of smoking, with reported bootstrap-corrected concordance index of 0.644 (22), which has been analogized to being marginally better than a coin toss in predicting benign tumors (20).

Angiomyolipomas and ROs accounted for two-thirds of our benign resections. We believe that the attempts to reduce benign resection should primarily be directed at nonextirpative diagnosis of these lesions, particularly when the renal mass under investigation demonstrates no obvious malignant features on imaging, such as infiltration of pelvicalyceal system, vessels, or sinus or perinephric fat.

Historically, a renal mass biopsy was not favored due to its invasive nature and fear of complications related to hemorrhage from the kidney or tumor and needle-tract seedling (24, 25). Other concerns were high nondiagnostic rates and sampling error due to tumor heterogeneity (24, 26). Contemporary data indicates a positive predictive value of 99.8%, nondiagnostic rates of 14%, complication rate of <5%, and exceedingly rare occurrence of needle-tract seeding (25, 27). Few other organs are removed without a biopsy diagnosis of malignancy; yet in a survey of 1131 urologists, 32% stated that they would never perform a biopsy for an SRM with 68% reporting that performing a biopsy would not change management (28), indicating that widespread adoption in clinical management algorithms is unlikely soon.

Advanced imaging technologies hold great promise in the noninvasive characterization of renal tumors. Although classic AMLs can be diagnosed on CT or MRI using well-established criteria, 5% of AMLs are fat-poor and cannot be differentiated from RCC on conventional imaging (29, 30). The use of multiparametric MRI in differentiating these lesions has been reported to have a sensitivity and specificity of 83 and 93%, respectively (31).

Molecular imaging entails the use of a radiopharmaceutical directed at a specific target protein or cellular process in combination with conventional cross-sectional imaging. ^99m^Tc-sestamibi accumulates in mitochondria, which are abundant in RO, and sparse in RCC. ^99m^Tc-sestamibi SPECT/CT takes advantage of this property with a reported sensitivity of 87.5% and specificity of 95.2% for the diagnosis of RO and hybrid oncocytic/chromophobe tumors (32).

The use of an artificial intelligence (AI) platform to analyze radiomics from cross-sectional imaging studies has been recently investigated with encouraging results (33). Radiomic and clinical variables of interest analyzed using a machine learning predictive model could differentiate benign versus RCC with an area under the receiver-operating characteristic curve of 0.84. In their study cohort of 684 patients, the authors estimated that the number of unnecessary surgeries for benign tumors could have been reduced from 15 to 3.5% by the use of AI to complement clinical data.

A third of our benign resections were non-AML/RO lesions. Current imaging technology cannot distinguish these lesions, and the diagnostic accuracy of percutaneous biopsy is unknown. With a nondiagnostic biopsy, particularly if the tumor is characterized as an SRM, active surveillance may be an option with delayed intervention for tumors that demonstrate growth (34, 35). In larger, symptomatic lesions or when a percutaneous biopsy indicates a tumor with uncertain malignant potential, an extirpative pathologic diagnosis is likely the safest option.

Our study is limited by its retrospective nature, with analysis based on data captured by our tumor registry. Not all desirable variables such as BMI, comorbidity, and nephrometry scores, among others, were recorded, hence were not available for analysis. Not all imaging studies were available for review. As mentioned earlier, our center, being an apex cancer institute, has distinctive referral patterns, hence our data may not be representative of data from other high-volume institutes or community.

## Conclusions

Our institutional data suggests a lower BKTRR compared to the published international literature, with no increasing trend. It is important to note that despite our significant referral bias, we were not immune to inadvertent benign resections, and our rates are likely to represent the lower limit of benign resections in the community. Population-based studies are needed to estimate the true prevalence of potentially unnecessary surgeries for benign kidney tumors.

## Figures and Tables

**Table S1:** Multivariate analysis for benign resection.

Variable	Odds ratio	95 % CI	P
Year of surgery	0.921	0.841–1.008	0.075
Age	0.987	0.947–1.028	0.522
Sex (male vs female)	0.550	0.188–1.609	0.275
Laterality (right vs left)	0.564	0.193–1.651	0.296
Tumor size	1.024	0.905–1.159	0.707
Incidental vs symptomatic	0.530	0.163–1.724	0.291
Hematuria vs no hematuria	6.411	0.765–53.698	0.087
Partial vs Radical nephrectomy	0.697	0.152–3.196	0.642
